# CRACM3 regulates the stability of non-excitable exocytotic vesicle fusion pores in a Ca^2+^-independent manner via molecular interaction with syntaxin4

**DOI:** 10.1038/srep28133

**Published:** 2016-06-15

**Authors:** Shuang Liu, Muhammad Novrizal Abdi Sahid, Erika Takemasa, Takeshi Kiyoi, Miyuki Kuno, Yusuke Oshima, Kazutaka Maeyama

**Affiliations:** 1 Department of Pharmacology, Ehime University Graduate School of Medicine, Shitsugawa, Toon-shi, Ehime 791-0295, Japan; 2 Department of Bioscience, Integrated Center for Sciences, Ehime University, Shitsukawa, Toon-shi, Ehime, 791-0295, Japan; 3 Department of Molecular and Cellular Physiology, Osaka City University Graduate School of Medicine, 1-5-7 Asahimachi, Abeno-ku, Osaka 545-8585, Japan; 4 Department of Molecular Medicine for Pathogenesis, Ehime University Graduate School of Medicine, Toon, Ehime 791-0295, Japan

## Abstract

Ca^2+^ release-activated calcium channel 3 (CRACM3) is a unique member of the CRAC family of Ca^2+^-selective channels. In a non-excitable exocytosis model, we found that the extracellular L3 domain and the cytoplasmic C-terminus of CRACM3 interacted in an activity-dependent manner with the N-peptide of syntaxin4, a soluble N-ethylmaleimide-sensitive factor attachment receptor protein. Our biochemical, electrophysiological and single-vesicle studies showed that knockdown of CRACM3 suppressed functional exocytosis by decreasing the open time of the vesicle fusion pore without affecting Ca^2+^ influx, the activity-dependent membrane capacitance (*Cm*) change, and the total number of fusion events. Conversely, overexpressing CRACM3 significantly impaired cell exocytosis independent of Ca^2+^, led to an impaired *Cm* change, decreased the number of fusion events, and prolonged the dwell time of the fusion pore. CRACM3 changes the stability of the vesicle fusion pore in a manner consistent with the altered molecular expression. Our findings imply that CRACM3 plays a greater role in exocytosis than simply acting as a compensatory subunit of a Ca^2+^ channel.

As a keystone of cell communication, regulated exocytosis, as well as constitutive secretion, is performed in certain excitable cells (e.g., neurons and endocrine, and exocrine cells) and non-excitable cells (e.g., mast cells and some other inflammatory cells). The fusion of vesicle membranes to the plasma membrane is required for the process of exocytosis. Alterations in the amount, location, and kinetics of release vesicles have profound consequences on the physiological functions of those cells. The dynamic capabilities of exocytosis are determined in part by the number of active vesicles, the dwell times of fusion pores, and the rate of vesicle recycling^1^. Membrane fusion during exocytosis and endocytosis relies on a core family of soluble N-ethylmaleimide-sensitive factor attachment receptor proteins (SNAREs). Syntaxin4 is a SNARE protein that is located on the target membrane. The interaction between syntaxin4 and other SNARE members on the vesicle membrane is thought to promote membrane fusion during exocytotic processes^2^. Active vesicles accumulate in the cytoplasm and undergo fusion only in response to the appropriate signal: occupation of plasma membrane receptors or depolarization accompanied by an increase in the intracellular calcium concentration ([Ca^2+^]_i_)^3^. Studies of excitable nerve terminals have demonstrated that the increase in membrane capacitance relies on steep increases in the presynaptic [Ca^2+^]_i_. At least four Ca^2+^ ions must bind to activate synaptic vesicle fusion, and half saturation occurs at 194 μM. If the [Ca^2+^]_i_ increases above 100 μM, synaptic vesicle exocytosis can occur within a few hundred microseconds^4^.

In most non-excitable cells, such as lymphocytes and mast cells, store-operated Ca^2+^ release-activated Ca^2+^ (CRAC) channels function as an essential route for Ca^2+^ entry. CRAC channels, also known as *ORAI*, are activated through the binding of the endoplasmic reticulum Ca^2+^ sensor stromal interaction molecule (STIM) to the CRAC channel proteins CRACM1, CRACM2 and CRACM3, of which CRACM1 is the major pore-forming subunit of the CRAC channel^5^,^6^. CRACM3 expression displays a tissue distribution at least as wide as that of CRACM1, whereas CRACM3 mRNA is much less expressed than CRACM1 in mammalian cells. Although CRACM1 and CRACM2 thus far appear to have very similar properties, CRACM3 differs in many ways and may be linked to specific functions. Nevertheless, CRACM3 is generally considered to be compensatory for the loss of CRACM1.

In the present study, we dissected the role of CRACM3 in the molecular interaction network of non-excitable exocytotic vesicle fusion, which is generally considered a distinct signal-transduction pathway from that of store-operated Ca^2+^ entry, with intracellular Ca^2+^ playing an intermediary role between them. Our biochemical, electrophysiological and single-vesicle monitoring results suggest that CRACM3 plays a role in the membrane-vesicle fusion process that is distinct from its function as a compensatory CRAC channel subunit.

## Results

### The molecular interaction of syntaxin4 and CRACM3 in a non-excitable vesicle fusion model

**Figure 1:**
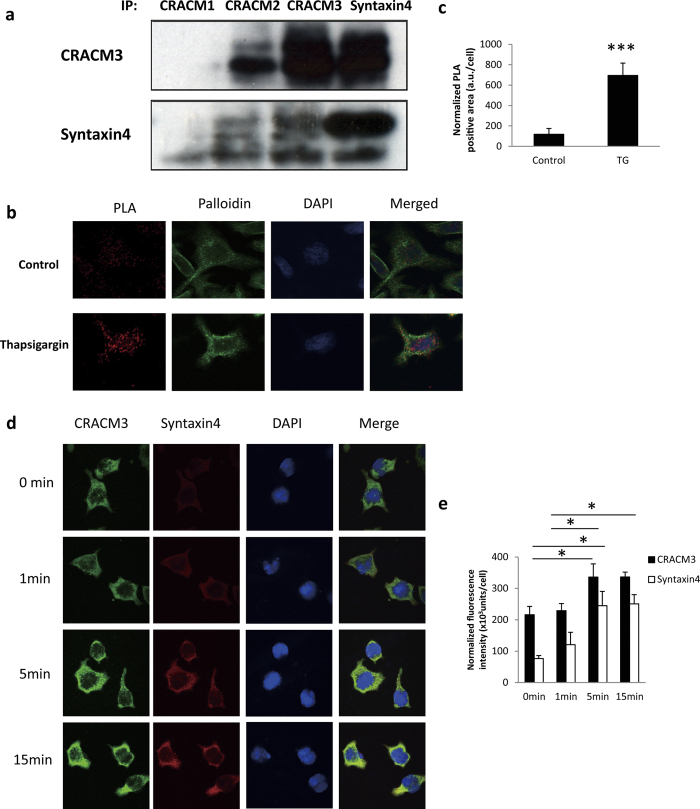
Molecular interaction of syntaxin4 and CRACM3 in RBL-2H3 cells. (**a)** CRACM3 and syntaxin4 protein levels were measured by western blotting after immunoprecipitation using specific antibodies for CRACM1, CRACM2, CRACM3, and syntaxin4. Full-length blots are presented in Supplementary Fig. 1. Upper: expression of CRACM3 in TG-stimulated cells (lane 1–4). Lower: expression of syntaxin4 in TG-stimulated cells (lane 1–4). **(b)** *In situ* PLA assay in non-stimulated cell and TG-stimulated cells. TG-stimulated or non-stimulated RBL-2H3 cells were used for *in situ* PLA analysis using a Duolink *In Situ* Detection Reagents Far Red detection kit. The slides were examined by confocal microscopy. The imaging data were analysed with Duolink ImageTool. Upper: Red PLA fluorescence signal, phalloidin green fluorescence signal, DAPI fluorescence signal and merged image in non-stimulated control cells. Lower: Red PLA fluorescence signal, phalloidin green fluorescence signal, DAPI fluorescence signal and merged image in TG-stimulated control cells. (**c)** Positive PLA area in non-stimulated and TG-stimulated cells normalized to DAPI fluorescence (n = 42, ***P < *0.001*). (**d)** The expression of CRACM3 and syntaxin4 at 0 min, 1 min, 5 min, 15 min in TG-stimulated cells detected by confocal microscopy. (**e)** Normalized fluorescence signals of CRACM3 and syntaxin4 using DAPI fluorescence (n = 40, *P < 0.05). Cells were examined by confocal microscopy, and imaging data were analysed with ImageJ software.

While studying SNARE proteins expressed in a rat basophilic leukaemia (RBL-2H3) cell line, a commonly used non-excitable exocytosis model that allows exocytosis to be easily monitored via secretory histamine release, we noticed that the CRACM3 signal appeared to be enriched in syntaxin4-immunoprecipitated blots. As shown in Fig. 1a and Supplementary Fig. 1a, proteins were immunoprecipitated from purified membrane protein fractions pooled from unstimulated or stimulated RBL-2H3 cells using specific antibodies and analysed to determine the expression of CRACM3 and syntaxin4. Thapsigargin (TG), a sarco/endoplasmic reticulum Ca^2+^-ATPase pump inhibitor, was applied to passively deplete Ca^2+^ stores and activate CRAC channels, triggering the vesicle fusion cascade for exocytosis. Upon syntaxin4 immunoprecipitation, blotting for CRACM3 revealed extremely high levels of CRACM3 amount following TG stimulation. The signal of syntaxin4 measured on CRACM3-immunoprecipitated blots was also very strong. These results strongly suggest an interaction between CRACM3 and syntaxin4.

To confirm the activity-dependent CRACM3-syntaxin4 interaction in RBL-2H3 cells, we performed an *in situ* proximity ligation assay (PLA). PLAs are a well-proven method to indicate close proximity in cells^7^. TG (0.5 μM) was used to trigger exocytosis. According to PLA methods, primary antibodies for syntaxin4 and CRACM3 were added, after which the PLA probes were bound. The hybridisation of the oligonucleotide arms of the PLA probes creates a template for rolling circle amplification only when the epitopes of the syntaxin4 and CRACM3 probes are in close proximity (<40 nm). The amplification products were labelled by FarRed, and the resulting spots of red fluorescence can be visualised under high power magnification. Red PLA fluorescence signals were detected in TG-stimulated cells, suggesting that CRACM3 co-localizes with syntaxin4 in activated RBL-2H3 cells. In contrast, red fluorescence signals were rarely detected in non-stimulated control cells (Fig. 1b,c). These results suggest that CRACM3 specifically co-localizes with syntaxin4 in an activity-dependent manner.

Given the differential compartmentation of syntaxin4 and CRACMs in inactivated and activated cells, we performed a time-course assay to detect the expression of these proteins after TG-stimulation (Fig. 1d,e). Both the syntaxin4 (green fluorescence) and CRACM3 (red fluorescence) signals gradually increased after TG-stimulation, suggesting the co-localization of these membrane proteins during exocytotic vesicle fusion. We noticed that the increase in CRACM3 protein levels was lower in the immunofluorescence analysis than in the western blotting results after immunoprecipitation. This difference is likely because immunofluorescence analysis does not capture the entire cell. The detection time points after TG-stimulation also differed between the experiments, namely, 30 min for western blotting and 15 min for immunofluorescence.

### Extracellular loop (L) 3 and the C-terminus of CRACM3 are critical for binding the N-peptide of syntaxin4

To elucidate the possible binding/interaction sites on CRACM3 and syntaxin4, a series of CRACM3 and syntaxin4 truncations was systematically generated. The N-terminal, C-terminal, and intracellular loops of His-tagged CRACM3 were incrementally removed up to the predicted membrane boundary of the TM segments (Supplementary Fig. 2a). For the generation of His-tagged syntaxin4 truncations, deletions of the predicted membrane-anchored SNARE helix (H3), interdomain-interaction region (IIS), and N-peptide region were prepared (Supplementary Figs 2b and 3). In RBL-2H3 cells, the interaction between endogenous CRACM3 and syntaxin4 was reproducible via exogenous expression of the wild-type His-tagged proteins in a pull-down assay. His-tagged CRACM3 failed to bind to the co-transfected wild-type syntaxin4 in HEK293A cells (data not shown), most likely due to the lack of other essential signalling components.

**Figure 2:**
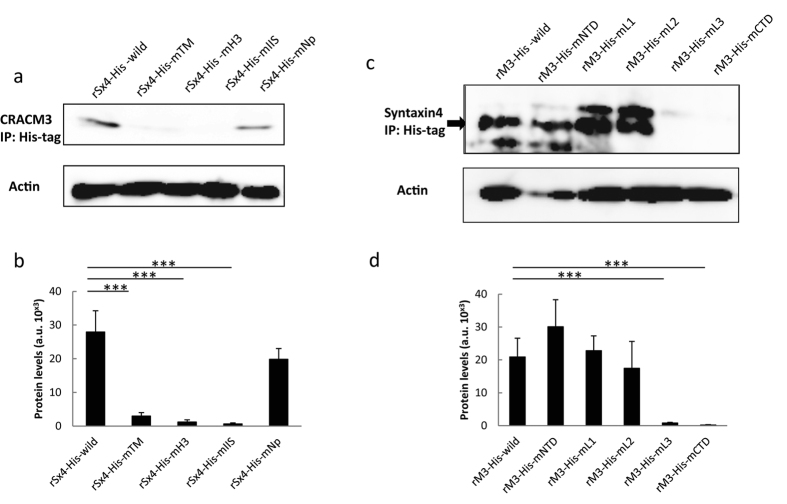
Extracellular loop (L) 3 and C-terminal domains of CRACM3 are critical for binding the syntaxin4 N-peptide. All plasmids were transfected into cells using RBL-2H3 Cell Avalanche^TM^ Transfection Reagent. At 72 h after transfection, the cells were stimulated with 0.5 μM TG for 15 min, and His-tag immunoprecipitation followed by western blotting using specific antibodies was performed. (**a)** CRACM3 protein, which was co-immunoprecipitated using the His-tag in rSx4-His-wild- (lane 1), rSx4-His-mTM- (lane 2), rSx4-His-mH3- (lane 3), rSx4-His-mIIS- (lane 4), and rSx4-His-mNp-transfected cells (lane 5), was detected using western blotting. Full-length blots are presented in Supplementary Fig. 1. Upper: CRACM3 expression in plasmid-transfected cells. Lower: CRACM3 expression normalized against actin levels in total cell lysates. (**b)** The protein levels of CRACM3 in His-tagged CRACM3 truncation-transfected cells after His-tag immunoprecipitation. (a.u., arbitrary area units; ***P < *0.001*; n = 3). (**c)** Syntaxin4 protein, which was co-immunoprecipitated with His-tag in rM3-His wild- (lane 1), rM3-His-mNTD- (lane 2), rM3-His-mL1- (lane 3), rM-His-mL2- (lane 4), rM3-His-mL3- (lane 5), and rM3-His-mCTD-transfected cells (lane 6), was detected by western blotting. Full-length blots are presented in Supplementary Fig. 1.Upper: syntaxin4 expression in the plasmid-transfected cells. Lower: syntaxin4 expression normalized against actin levels in total cell lysates. (**d)** The protein levels of syntaxin4 in His-tagged CRACM3 truncation-transfected cells after His-tag immunoprecipitation. (a.u., arbitrary units; *** *P* < *0.001*; n = 3).

After TG stimulation, CRACM3 was co-immunoprecipitated with wild-type His-tagged syntaxin4 (rSx4-his-wild), as well as with a syntaxin4 truncation that included the N-peptide (rSx4-his-mNp), but not with an H3-including truncation (rSx4-his-mH3) or an IIS-including truncation (rSx4-his-mIIS) (Fig. 2a,b and Supplementary Fig. 1c). These results indicate that the N-peptide is necessary for syntaxin4 to bind CRACM3.

Syntaxin4 was detected in His-tag immunoprecipitations from cells transfected with wild-type His-tagged CRACM3 (rM3-his-wild), as well as from cells transfected with His-tagged CRACM3 mutants lacking the N-terminal (rM3-his-mNTD), L1 (rM3-his-mL1), or L2 (rM3-his-mL2) domains, but not from cells transfected with mutants lacking the L3 (rM3-his-mL3) or C-terminal (rM3-his-mCTD) domains (Fig. 2c,d and Supplementary Fig. 1d). The results indicate that interaction sites for the syntaxin N-peptide are likely to be located in the C-terminal domain and extracellular loop 3 of CRACM3.

### CRACM3 regulates non-excitable exocytosis independent of store-operated Ca^2+^ entry (SOCE)

**Figure 3:**
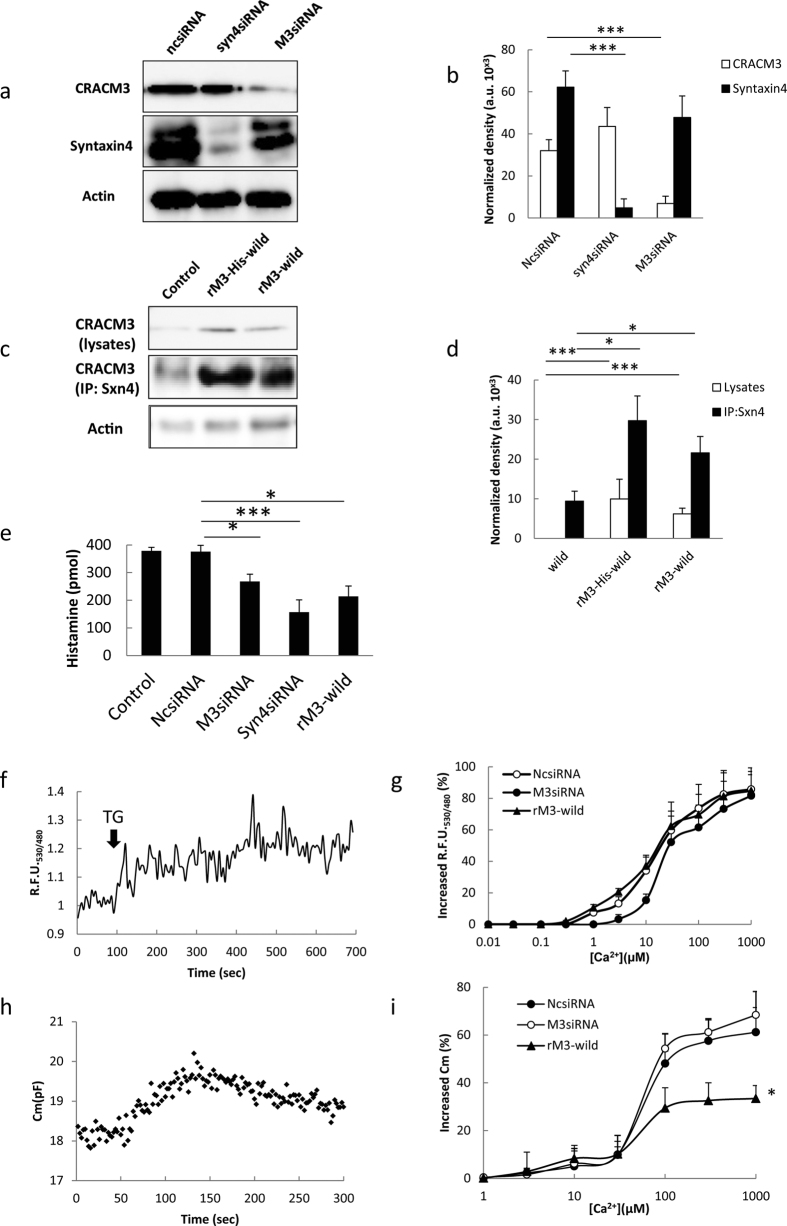
CRACM3 regulates non-excitable exocytosis in a SOCE-independent manner. **(a)** The protein levels of CRACM3 and syntaxin4 3 d post transfection of siRNA were detected by western blotting following siRNA-mediated protein suppression. Upper: CRACM3 expression in NCsiRNA- (lane 1), Syn4siRNA- (lane 2), and M3siRNA-transfected cells (lane 3). Middle: syntaxin4 expression in each group of cells. Lower: actin expression in each group of cells. **(b)** CRACM3 and syntaxin4 expression normalized against actin levels (a.u., arbitrary units; ***P < *0.001*; n = 3). The samples derive from the same experiment and that blots were processed in parallel. **(c)** Overexpression of CRACM3 was observed in rM-wild-transfected RBL-2H3 cells. CRACM3 expression was detected by western blotting 10 d post transfection. Upper: CRACM3 expression in total cells lysates of control cells (lane 1), rM3-His-wild-transfected cells (lane 2), and rM3-wild-transfected cells (lane 3). Middle: CRACM3 expression detected by western blotting followed by syntaxin4 immunoprecipitation in each group of cells. Lower: actin expression in the total cell lysates of each group of cells. **(d)** CRACM3 expression in total cells lysates and after syntaxin4 immunoprecipitation normalized against actin levels (a.u., arbitrary unit; *P < *0.05*; ***P < *0.001*; n = 3). The samples derive from the same experiment and that blots were processed in parallel. **(e)** Typical Ca^2+^ influx patterns in cameleon (YC4.60)-transfected cells. The assay was performed using a fluorometric imaging plate reader, and the results are presented as the ratio of relative fluorescence units (R.F.U. 530 nm/480 nm). **(f)** Extracellular Ca^2+^ titration curve of M3siRNA-transfected, NCsiRNA-transfected, and rM3-wild-transfected cells (n = 8–10) that were co-transfected with YC4.60. The data are presented as the means ± s.e.m. **(g)** A typical *C*_*m*_ curve was measured under whole-cell clamp conditions, and the change in *C*_*m*_ was triggered by 3 μM [Ca^2+^]_free._ **(h)** The [Ca^2+^]_i_-dependence of the capacitance responses (Δ*Cm*) was observed in M3siRNA-transfected, NCsiRNA-transfected, and rM3-wild-transfected cells (*P < *0.05*; n = 25–40 individual cells per group). The data are presented as the means ± s.e.m. **(i)** The level of released histamine in control cells, NCsiRNA-transfected cells, M3siRNA-transfected cells, and rM3-wild-transfected cells (*P < *0.05*; ***P < *0.001*; n = 6). The results are expressed as the mean ± s.e.m. All full-length blots are presented in Supplementary Fig. 1.

The molecular interaction between a CRAC channel and SNARE protein allow us to tackle the question of whether CRACM3 might have a specific function in the exocytotic network. Specific alterations in protein expression were used to investigate the contribution of CRACM3 to exocytosis. To suppress protein expression, CRACM3 or syntaxin4 gene-silencing was performed using specifically designed short interfering RNAs (siRNAs) against CRACM3 (M3siRNA) and syntaxin4 (Syn4siRNA). Significant decreases were observed in the expression of CRACM3 or syntaxin4 in RBL-2H3 cells on the third day after transfection with the specific siRNAs (Fig. 3a,b and Supplementary Fig. 1e,f).

As a comparison, rSx4-wild-pcDNA3 or rM3-wild/rM3-his-wild-pcDNA3 were used to overexpress these proteins in RBL-2H3 cells. CRACM3 signals were detected in the cell lysates of rM3-wild- and rM3-his-wild-pcDNA3-transfected cells, whereas CRACM3 was almost undetectable in control cell lysates. Overexpression of CRACM3 caused a significant increase in the amount of co-immunoprecipitated CRACM3 that could be pulled down with a syntaxin4 antibody compared with that in the control cells (Fig. 3c,d and Supplementary Fig. 1g,h). However, because the overexpression of syntaxin4 induced extensive apoptosis in RBL-2H3 cells, a stable syntaxin4-overexpression model could not be established (data not shown). There were no significant differences in CRACM1 protein levels in ncsiRNA-, M3siRNA-, and rM3-wild-transfected cells (Supplementary Fig. 4).

The release of histamine, a hallmark of functional exocytosis in activated RBL-2H3 cells, was evaluated in the CRACM3-knockdown, CRACM3-overexpression, and syntaxin4-knockdown cells (Fig. 3e). There was no difference in histamine release between control cells and negative control siRNA (NCsiRNA)-transfected cells. Compared with the NCsiRNA-transfected group, the level of histamine release decreased to 71.3% (*P* < 0.05) in M3siRNA-transfected cells and 41.9% (*P* < 0.001) in Syn4siRNA-transfected cells, suggesting that knocking down either CRACM3 or syntaxin4 inhibits functional exocytosis in RBL-2H3 cells. CRACM3 overexpression did not promote exocytosis in rM3-wild-transfected cells. Instead, we observed an unexpected, 56.9% reduction in histamine release (*P* < 0.05). Thus, both the knockdown and overexpression of CRACM3 can suppress functional non-excitable exocytosis in RBL-2H3 cells.

The suppression of exocytosis by knocking down syntaxin4 is expected because syntaxin4 is a key member of the membrane SNARE family^8^. Without syntaxin4 forming the SNARE complex, vesicles are not able to undergo plasma membrane fusion, which is indispensable for exocytosis. Therefore, what is the role of CRACM3 in cell exocytosis? One simple explanation is that CRACM3 might perform a role in exocytosis via the [Ca^2+^]_i_ because CRACM3 is generally considered to be a CRAC channel subunit.

Does altered CRACM3 expression disrupt CRAC channel function? To test this possibility, we monitored Ca^2+^ influx through CRAC channels in M3siRNA-transfected, NCsiRNA-transfected, and rM3-wild-transfected cells using a low-affinity Ca^2+^ indicator, cameleon (YC4.60) (minimum Ca^2+^ sensitivity of approximately 10^−5^, *K*_*d*_ = 14.4 μM); this indicator was localized just beneath the plasma membrane and responds to a relatively strong Ca^2+^ influx through CRAC channels on the plasma membrane but not intracellular Ca^2+^ oscillations (Fig. 3f). The extracellular Ca^2+^ titration curve of YC4.60 in M3siRNA-transfected cells was slightly right-shifted compared with that in NCsiRNA-transfected cells. This shift was not significant (*P* = 0.86), with *K*_*m*_ = 27.97 μM for M3siRNA-transfected cell and *K*_*m*_ = 15.01 μM for NCsiRNA-transfected cells (Fig. 3g). Overexpression of CRACM3 did not affect the titration curve of rM3-wild-transfected cells (*K*_*m*_ = 10.91 μM). The results of the whole-cell recordings also did not show any difference in the maximal responses induced by TG in M3siRNA-, NCsiRNA-, and rM3-wild-transfected cells (Supplementary Fig. 5). These results suggest that the overexpression or silencing of CRACM3 does not have a significant impact on SOCE.

Because the effect of CRACM3 on exocytosis is Ca^2+^ independent, the next mechanistic possibility is that CRACM3 directly regulates exocytotic fusion to alter release capability. Thus, we evaluated changes in the plasma membrane capacitance (*C*_*m*_), which is triggered by different [Ca^2+^]_i_, in M3siRNA-, NCsiRNA-, and rM3-wild-transfected RBL-2H3 cells. Under whole-cell patch clamp, the relative change in *C*_*m*_ (Δ*C*_*m*_), which is caused by changes in the cell surface area due to vesicle fusion to the membrane, was monitored in response to cytosol dialysis using a pipette solution containing different concentrations of intracellular free Ca^2+^ ([Ca^2+^]_free_). A typical *C*_*m*_ curve generated using 3 μM [Ca^2+^]_free_ is shown in Fig. 3h. The [Ca^2+^]_i_ dependence of the capacitance responses was observed in M3siRNA-, NCsiRNA-, and rM3-wild-transfected cells (Fig. 3h). The concentration-response data are best described by a Richard’s five-parameter equation with a slope coefficient of 1.0, a *LogEC*_*50*_ of 72.65 μM, an *S* of 0.5, and a maximal Δ*C*_*m*_ of 68.02% in M3siRNA-transfected cells; in contrast, NCsiRNA-transfected cells had a slope coefficient of 1.0, a *LogEC*_*50*_ of 79.75 μM, an *S* of 0.5, and a maximal Δ*C*_*m*_ of 61.23% (Fig. 3i). Hence, a defect in CRACM3 expression does not affect the Δ*C*_*m*_ at a predefined [Ca^2+^]_free._ In rM3-wild-transfected cells, the slope of the concentration-response curve was obviously different than that in NCsiRNA-transfected cells, with a slope coefficient of 0.59, a *LogEC*_*50*_ of 47.15 μM, an *S* of 0.31, and a significantly decreased maximal Δ*C*_*m*_ of 33.52%.

Taken together, our results show that altering CRACM3 expression does not affect the intracellular Ca^2+^ pathway. Defective CRACM3 down-regulates histamine release but has no influence on Δ*C*_*m*_, whereas CRACM3 overexpression reduces the dynamic capabilities of exocytosis.

### CRACM3 affects cell exocytosis by influencing the activity-dependent fusion pore open time

Real-time exocytotic vesicle dynamics were monitored using single-nanoparticle-containing vesicles in M3siRNA-, NCsiRNA-, and rM3-wild-transfected cells. Single vesicles were efficiently labelled through the use of streptavidin-coated Qdots conjugated to biotinylated antibodies against the luminal domain of the vesicular protein synaptotagmin, modified from a published design^9^. To report fusion events on the cell membrane, Qdots with a diameter of ~18 nm provide a suitable artificial cargo that is small enough to fit into the vesicular lumen and also large enough to remain trapped inside vesicle fusion pores (pore diameter: <10 nm; vesicle diameter: ~50–800 nm)^10^. Qdot photoluminescence was pH dependent and increased when the pH was raised from 5.48 (intravesicular) to 7.34 (extracellular). The pH-dependence predicts distinct patterns of Qdot photoluminescence upon vesicle fusion: rapid retrieval of the fusion pore would allow protons, but not the Qdot, to escape, causing transient deacidification and Qdot brightening. A stable fusion pore would appear as a similar Q-dot brightening followed by the loss of signal as the Qdot departs^11^.

**Figure 4:**
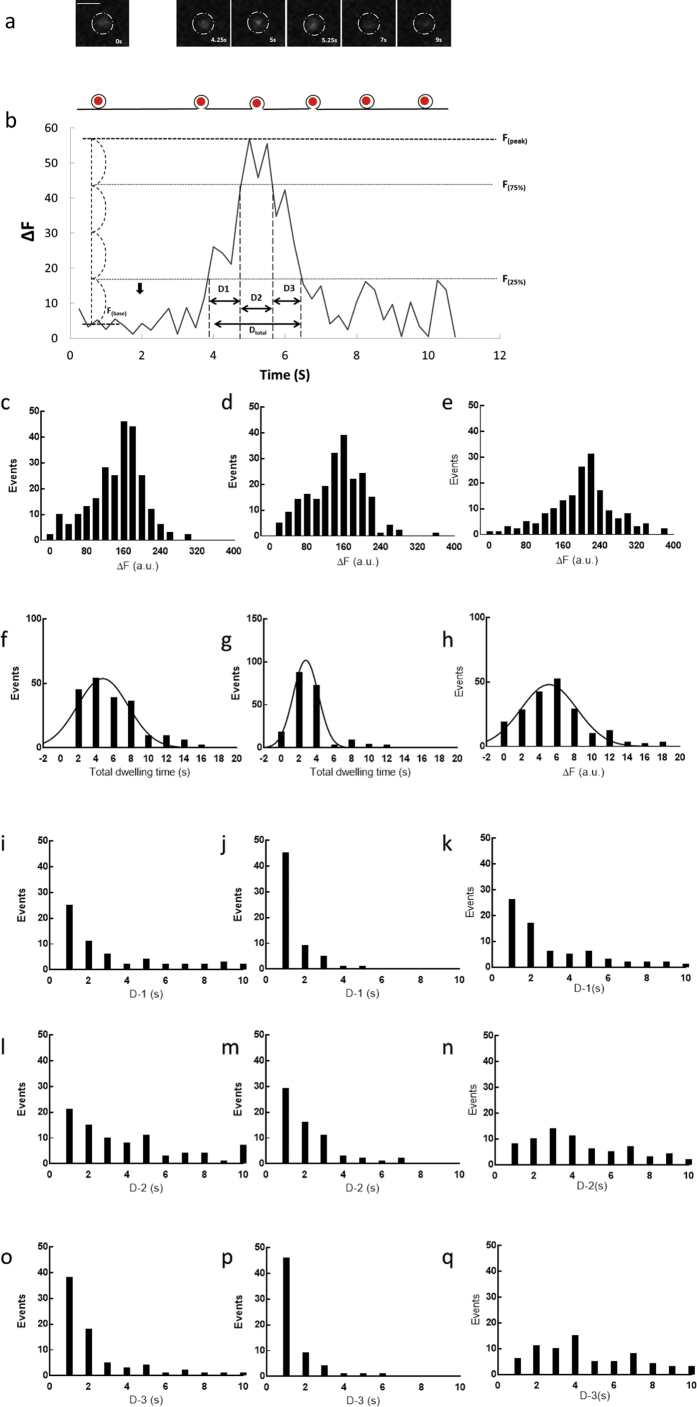
CRACM3 affects exocytosis through its influence on the activity-dependent fusion pore open time. Single vesicles were efficiently labelled with streptavidin-coated Qdots conjugated to biotinylated antibodies against the luminal domain of the vesicular protein synaptotagmin. **(a)** The Qdot-loaded plasma membrane exhibited different patterns of photoluminescence upon TG-stimulation, including baseline noise and a transient positive deflection (uptick). The uptick level is defined as a distinct peak ~25% above baseline that is distinct from the transient baseline noise. The upticks can be attributed to an initial state, a fully open state, and a closed state. **(b)** Images of a Qdot cluster at multiple time points, taken with a custom-designed upright multi-photon excitation microscopy system. White scale bar: 1.5 μm. **(c**–**e)** The histogram of photoluminescence for 250 single stimulus presentations in NCsiRNA-transfected (**c**), M3siRNA-transfected (**d**), and rM3-wild-transfected cells (**e**). **(f**–**h)** Histogram of the total dwell time of 60 vesicles in NCsiRNA-transfected (**f**), M3siRNA-transfected (**g**), and rM3-wild-transfected cells (**h**). The smooth curve is the fit of the Gaussian distribution. **(i–k)** Distribution of times of initial states (D-1) in NCsiRNA-transfected (**i**), M3siRNA-transfected (**j**), and rM3-wild-transfected cells (**k**). **(l**–**n)** Distribution of times of full-open states (D-2) in NCsiRNA-transfected (**l**), M3siRNA-transfected (**m**), and rM3-wild-transfected cells (**n**). **(o**–**q)** Distribution of times of closed states (D-3) in NCsiRNA-transfected (**o**), M3siRNA-transfected (**p**) and rM3-wild-transfected cells (**q**).

The Qdot-loaded plasma membrane exhibits different patterns of photoluminescence upon TG stimulation (1 μM), including baseline noise and a transient positive deflection (uptick). The uptick level is defined as a distinct peak ~25% above baseline that is distinct from the transient baseline noise (Fig. 4a). The upticks can be attributed to three phases: i) an initial state immediately after fusion opening, in which the photoluminescence increases rapidly from ~25% to 75% above baseline noise; ii) a full-open state, in which the photoluminescence remains at the maximal value until decreasing to ~75% above baseline noise; iii) a closed state, wherein the photoluminescence decreases to ~25% above baseline noise until returning to baseline level, and then, in some cases, decays (Fig. 4a,b and Supplementary Fig. 6a–c).

An assessment of the relative increase in photoluminescence (ΔF) showed no significant difference in the number of total vesicle fusion events with distinguishable upticks in M3siRNA- and NCsiRNA-transfected RBL-2H3 cells. These results support our electrophysiological results that no changes in Δ*C*_*m*_ occur in CRACM3-silenced cells. The total number of exocytotic events was much lower in rM3-wild transfected cells than in NCsiRNA-transfected and M3siRNA-transfected cells (Fig. 4c–e).

The distribution of total dwell times from the initial opening of the fusion pore to the end of the closed state is presented in Fig. 4f (NCsiRNA-transfected cells), 4g (M3siRNA-transfected cells) and 4h (rM3-wild-transfected cells). Smoothed curves are fit with a Gaussian distribution, yielding a mean of 3.05 s with a standard error of the mean (SEM) of 0.12 s in NCsiRNA-transfected cells, a mean of 2.1 s with a SEM of 0.08 s in M3siRNA-transfected cells (*P* < 0.05), and a mean of 3.72 s with a SEM of 0.11 s in rM3-wild-transfected cells (*P* < 0.05). Silencing CRACM3 reduced the total dwell time, whereas overexpression of CRACM3 significantly prolonged the total dwell time of the fusion pore.

The dwell times of the initial state, the full-open state, and the closed state were analysed by the chi-squared test in M3siRNA-, NCsiRNA-, and rM3-wild-transfected cells (Fig. 3i–q). The dwell times of the initial state (D-1) and the full-open state (D-2) were significantly shorter in M3siRNA-transfected cells than in NCsiRNA-transfected cells. D-1 was 0.93 s ± 0.13 s and D-2 was 1.81 s ± 0.31 s in M3siRNA-transfected cells (Fig. 4I,j); in contrast, NCsiRNA-transfected cells had a D-1 of 2.25 s ± 0.47 s (*P* < 0.05) and D-2 of 3.06 s ± 0.67 s (*P* < 0.05) (Fig. 4l,m). There was no significant difference between the dwell time of the closed state (D-3) in NCsiRNA- (1.47 s ± 0.65 s) and M3siRNA-transfected cells (1.24 ± 0.59 s) (Fig. 4o,p). Although no change in D-1 was observed between NCsiRNA- and rM3-wild-transfected cells, D-2 and D-3 were obviously prolonged in rM3-wild-transfected cells compared with that of NCsiRNA-transfected cells, with a D-2 of 3.98 s ± 1.02 s (*P* < 0.05) and a D-3 of 4.35 s ± 1.07 s (*P* < 0.05) (Fig. 4k,n,q, and Supplementary Fig. 6d).

Together, these results suggest that CRACM3 regulates exocytosis in non-excitable cells by influencing the activity-dependent fusion pore open time.

## Discussion

The field of agonist-activated Ca^2+^ entry in non-excitable cells was revolutionized approximately 10 years ago by the discovery of the CRAC family as the essential pore-forming components of these highly Ca^2+^-selective channels^12^,^13^; however, relatively few studies have focused specifically on CRACM3, and it has been called ‘the neglected’ or ‘the exceptional’ CRAC^13^. In the current study, we uncovered evidence that CRACM3 physically and functionally correlates with syntaxin4, a plasma membrane SNARE protein, during secretory granule exocytosis in a non-excitable cell model. CRACM3 changes the stability of the activity-dependent vesicle fusion pore in a manner consistent with the alteration of molecular expression.

Although CRACM3 has the ability to form heteromers, even with strong CRACM3 overexpression, CRACM1 is still essential for forming CRAC channels. The stoichiometry of human CRACM1 and CRACM3 was investigated using single-molecular photobleaching of tagged constructs. Whereas CRACM1 and CRACM3 were predominantly detected as dimers under resting conditions, they were detected as tetramers upon channel activation^13^. As the newest CRAC family member in evolutionary terms, CRACM3 shares only 57% amino acid sequence identity with CRACM1, with 46% in the C-terminal domain and 21% identity in the extracellular loop between transmembrane domains 3 and 4 (L3). CRACM3 lacks the ~18-amino-acid N-terminal hydrophilic stretch found in CRACM1, which together with the nearby proline-rich domain mediates reactivation after fast Ca^2+^-dependent inactivation^14^. CRACM3 has a long (72 amino acid) non-pore-forming extracellular L3 and a coiled-coil domain within the short cytoplasmic C-terminus. The presence of these marked differences in the CRACM3 sequence raises the possibility that these features may impart unique properties to channel regulation. Our immunoprecipitation results showed that the extracellular L3 domain and the cytoplasmic C-terminus of CRACM3 undergo an activity-dependent interaction with the syntaxin N-peptide, which is a positive regulator of SNARE assembly at the plasma membrane to facilitate membrane fusion.

In RBL-2H3 cells, four types of target-membrane SNAREs (SNAP23, syntaxin2, syntaxin3, and syntaxin4) can be detected at the protein level^8^. Syntaxin4, syntaxin3, and SNAP23, but not syntaxin2, are the t-SNARE proteins responsible for forming the SNARE complex and contribute to vesicle fusion^15^. Similarly to most other syntaxins, syntaxin4 contains a predicted membrane-anchored helix and a functional interdomain site that can bind to the H3 helix, forming a closed syntaxin conformation. The syntaxin4 N-peptide is a 19 amino acid long segment located within the cytoplasmic C-terminus. We compared the sequences of 40-amino-acid-long segments within the cytoplasmic C-termini of syntaxin3 and syntaxin4, which should contain the predicted N-peptide region. Synatxin3 shares only 32.0% amino acid sequence identity with syntaxin4 (Supplementary Fig. 7). Interactions between N-peptides and other accessory proteins on the plasma membrane play a positive regulatory role in SNARE assembly. Exocytosis via syntaxins involves a conformational switching from an ‘open’ (SNARE-complex compatible) form, which is ready to assemble the SNARE complex, to a ‘closed’ (SNARE complex-incompatible) form, while SNAREs and other accessory proteins accumulate on the pre-exocytotic plasma membrane. Then, the closed-form interaction would be ‘opened’ again for subsequent rounds of fusion^16^. During the conformation switch, a regulator protein, such as sec1/munc18, binds to a SNARE, and this binding involves an initial interaction via the N-peptide^17^. Structural work on neurotensin^18^, a G protein-coupled receptor with seven transmembrane helices (TM1-TM7), showed that this protein undergoes conformational changes that ultimately allow extracellular agonist binding and the activation of a G protein at the intracellular surface of the receptor. Specifically, an inward shift of the extracellular regions of TM2 and TM6 was observed, likely due to the pronounced kink at A120^2.57^ of TM2 and a change in the tilt of TM6, yielding a deep and narrow ligand-binding pocket, with residues from extracellular (ECL) 2, ECL3, TM6, and TM7 near the intracellular face providing ligand-receptor contacts. Although we do not yet understand the conformational change of CRACM3 upon TG-stimulation, we can hypothesize that the extracellular loop is inlaid into the plasma membrane as it is in SNARE complexes. In addition to the cytoplasmic C-terminus, the contribution of the extracellular L3 of CRACM3 to the interaction with the cytoplasmic syntaxin N-peptide is interesting and may indicate that CRACM3 binds syntaxin4 in the closed conformation because it could be inlaid into the plasma membrane as a result of the conformation change similarly to SNARE complexes. Our planned crystal structure studies will help to provide further insights into the structural features of the CRACM3-syntaxin4 interaction.

The activity-dependent molecular proximity of CRACM3 and syntaxin4 allows us to investigate the functional impact of CRACM3 on non-excitable exocytotic processes, which essentially require the assembly of SNARE proteins on the vesicle and plasma membranes. The capacity for functional exocytosis in non-excitable RBL-2H3 cells was monitored simply via the release of histamine from exocytotic vesicles, which was triggered by passive depletion of Ca^2+^ stores to activate CRAC channels. The significantly decreased levels of histamine release in both CRACM3-silenced and CRACM3-overexpressing cells suggest that CRACM3 likely contributes to the exocytotic processes. The modulation of exocytotic vesicle fusion modes depends on the interplay between the tendency to collapse and restraining mechanisms. A further distinction can be made between i) core players in the generation of forces, such as SNARE complexes and complexin; ii) direct or indirect regulators of these core players (e.g., Ca^2+^, synaptotagmin, and other interacting proteins); and iii) bystander elements that spare pro-fusion and restraining forces but otherwise influence the redistribution of membrane molecules while fusion is maintained^1^. In the present study, we propose that CRACM3 belongs in the latter two categories based on its direct interaction with syntaxin4 and its ability to modulate fusion pore stability.

The silencing of CRACM3 expression does not appear to influence the dose-response curve for [Ca^2+^]_free_-Δ*Cm*, according to the results of whole-cell capacitance recording, which reflect the membrane surface area and its increase upon fusion. This result was also corroborated by single-vesicle observation studies using exogenous quantum dots. CRACM3 silencing did not affect the number of total exocytotic events within a ~10-min recording period. However, significant decreases in the dwell times of the initial and full-open states were observed, which may explain why functional exocytotic capability is supressed in CRACM3-silenced cells. It is still controversial whether this rapid opening and closing fusion mode is exactly the same manner as kiss-and-run fusion in neuronal cells; however, similar exocytosis modes, in which vesicles release their contents through a transient, narrow fusion pore with a short fusion pore open time, have been observed in both excitable neuronal cells and endocrine cells^1^. Rapid retrieval of the fusion pore has been shown to have a number of consequences for the quantity and/or quality of information conveyed by secretory activity. In the present study, the output of histamine was suppressed by the alteration of the exocytosis mode by CRACM3 modulation. In adrenal chromaffin cells, the switch to the transient exocytosis mode appeared to coincide with the release of catecholamines while likely retaining the larger neuropeptides^19^. Thus, the exocytosis-mode switch induced by the siRNA-knockdown of CRACM3 may also allow the differential release of multiple cargos, thus expanding the sophistication of secretory signalling.

A distinct exocytosis mode, in which the fusion pore appears to exhibit prolonged open and closed states, was detected in the CRACM3-overexpressing cells. The significantly decreased number of fusion pores, along with the relatively long lasting time during which the photoluminescence remained above 75% of the basal level, suggests that the quantum dot became stuck in a partially open fusion pore while retaining its gross morphological shape. The existence of this mode suggests that the probability of either the vesicle fully flattening into the planer surface or being quickly retrieved would be much lower in CRACM3-overexpressing cells than in normal control cells. Along with the decreased histamine release, significant suppression of the activity-dependent Δ*Cm* and the number of total fusion events were observed. Overabundant CRACM3 irreversibly prevents the completion of vesicle-membrane fusion.

As described above, CRACM3 may directly interact with the syntaxin4 N-peptide in the ‘closed’ conformation. Furthermore, the silencing of CRACM3 expression did not change the total number of exocytotic events, which is initially regulated by the N-peptide. We hypothesize that CRACM3 modulates the conformational switch of syntaxin4 from the ‘closed’ conformation to the ‘open’ conformation, thus making it ready for subsequent rounds of fusion. The absence of CRACM3 decreased the open time of the fusion pore, whereas CRACM3 overexpression prolonged the stability of pore opening and hindered the exocytotic capability by inhibiting the retrieval of vesicles. Thus, CRACM3 must act as a negative input for the closed-open mode-switching of syntaxin4 to shape the final fusion event.

Overall, when modulating exocytotic vesicle fusion modes, CRACM3 appears to be both a direct regulator through its interaction with SNAREs on the plasma membrane and a bystander element that alters the balance of pro-fusion and restraining forces; this interaction is Ca^2+^-independent. Regulation by CRACM3 could be beneficial for modulating release selectivity and capability by controlling the opening of the transient fusion pore.

Researchers are increasingly realizing that CRACM3 possesses unique properties and behaves in unique ways that are distinct from either CRACM1 or CRACM2. In previous studies, neither the co-expression of CRACM3 together with STIM1 nor the siRNA-induced knockdown of CRACM3 generated any measurable increases in SOCE^20^,^21^. CRACM3 was reported to rescue Ca^2+^ signals only after knockdown of endogenous CRACM1 but had no effect in the presence of normal CRACM1^12^. In the present study, we monitored the Ca^2+^ signal just after influx across the plasma membrane and found that CRACM3 overexpression did not affect the *K*_*m*_ of the [Ca^2+^]-response titration curve and that siRNA-knockdown of CRACM3 only slightly shifted the *K*_*m*_ to the right, from 14.4 μM to 27.97 μM. The functional relevance of CRACM3 as a store-operated channel still remains speculative.

Although the question of whether CRACM3 is a bona fide ion channel by itself or whether it is just a compensatory subunit remains unresolved, CRACM3 does modulate activity more moderately in non-excitable cells than does CRACM1. We successfully suppressed inflammatory responses and autoimmune responses in a collagen-induced arthritis model with milder side effects via the systemic lentivirus-mediated delivery of shRNA-targeting CRACM3^22^. Silencing of CRACM3 by siRNA inhibited breast cancer cells, arrested the cell cycle at G1 and even reduced the expression and function of the c-myc proto-oncogene. Our study provides evidence that CRACM3 directly interacts with syntaxin4 located at the plasma membrane. CRACM3 is also capable of modulating exocytosis by changing the stability of the vesicle fusion pore and switching the fusion mode, which may enable the differential release of multiple secretory cargos. Although further investigation of the functional effect of CRACM3 on exocytotic release and post-exocytosis signalling, which is influenced by the vesicle fusion mode, is necessary prior to *in vivo* or clinical studies, we believe that the basic findings described in this article provide grounds for optimism regarding the application of CRACM3 regulators to diseases related to non-excitable cells.

## Methods

### Cell culture and transfection

RBL-2H3 cells were cultured in Eagle’s minimal essential medium containing 15% foetal calf serum at 37 °C in a humidified atmosphere with 5% CO_2_. The siRNA/siRNA cocktails used in the experiments are shown in Table 1. The oligonucleotides were transfected according to the manufacturer’s instructions using Oligofectamine^TM^ Transfection Reagent (Thermo Fisher, Waltham, MA, USA). Plasmids expressing rSx4-wild, rSx4-His-wild, rSx4-His-mTM, rSx4-His-mH3, rSx4-His-mIIS, rSx4-His-mNp, rM3-His wild, rM3-His-mNTD, rM3-His-mL1, rM-His-mL2, rM3-His-mL3, rM3-His-mCTD, and cameleon (pc4.60) were transfected into cells using RBL-2H3 Cell Avalanche^TM^ Transfection Reagent (EZ Biosystems, College Park, MD, USA) according to the manufacturer’s instructions. Cells were assayed 72 h after DNA or siRNA/siRNA cocktail transfection.

The amount of histamine released from cells was measured by high-performance liquid chromatography-fluorometry^2^. *O*-phthalaldehyde-labelled histamine was detected by fluorometry using an L-7480 FL-Detector (Hitachi, Tokyo, Japan).

### Immunoprecipitation and western blotting

Immunoprecipitation and western blotting were performed according to a previously described method^23^. Cells were lysed, and membrane proteins were extracted using a Mem-PERTM Plus Membrane Protein Extraction Kit (Thermo Scientific, Rockford, IL, USA) according to the manufacturer’s instructions. The protein concentration was determined using the Bio-Rad protein assay. A total of 3 μg of specific antibody was added to 300 μl of cell extracts and incubated for 1 h at 4 °C. Next, 30 μl of a 1:1 slurry of protein-G Sepharose 4B beads was added to the antibody-extract mix. After being washed, the beads were suspended in 30 μl of SDS loading buffer to release the bound proteins. Aliquots of this solution were loaded onto 12% denaturing polyacrylamide gels and transferred to a polyvinylidene difluoride transfer membrane. The blots were incubated with specific primary antibodies and horseradish peroxidase-conjugated secondary antibodies. The blots were then treated with a chemiluminescent substrate.

### *In situ* proximity ligation assay (PLA) and immunofluorescence analysis

*In situ* PLA is a technique capable of detecting protein interactions with high specificity and sensitivity^24^. RBL-2H3 cells were stimulated with or without TG and then used for *in situ* PLA analysis using a Duolink *In Situ* Detection Reagents Far Red detection kit (Olink Bioscience, Uppsala, Sweden). Briefly, cells were fixed on slides using an ice-cold acetone. After being blocked with 3% bovine serum albumin in phosphate-buffered saline, the primary antibodies rabbit polyclonal anti-syntaxin4 IgG (SIGMA-ALDRICH, St. Louis, MO, USA) and monoclonal anti-CRACM3 IgG (clone 2H2G9, Abnova, Taiwan) were added to the slides. After washing steps, the two PLA probes (anti-mouse MINUS and anti-rabbit PLUS) were added to the slides, and the slides were then incubated in a humidified chamber for 1 h at 37 °C. Following a 30-min incubation with ligation-ligase solution, the slides were treated with amplification-polymerase solution. Then the slides were stained by an Acti-stain^TM^ 488 Fluorescent Phalloidin reagent (Cytoskeleton, Denver, CO, USA) for 30 min at room temperature. The slides were finally mounted in media containing 4′6-diamidino-2-phenylindole (DAPI) and examined by confocal microscopy using a Nikon C1 microscope (Nikon, Tokyo, Japan) at the same settings to allow for quantitation. The captured imaging data were analysed with the Duolink ImageTool (Cytoskeleton), which is specifically designed for the objective quantification of PLA signals in images. The PLA signal is recognized as a spot, and an individual signal is of submicrometer size. The nuclei are automatically detected, and the cytoplasm size is estimated, enabling the single-cell statistical analysis of expression levels in cell populations.

For immunofluorescence analysis, cells were fixed at 0 min, 1 min, 10 min, and 15 min post stimulation using a 50:50 ice-cold methanol/acetone. After being blocked, the cells were incubated with a mixture of rabbit polyclonal anti-syntaxin4 IgG and monoclonal anti-CRACM3 IgG. Alexa Fluor 488-conjugated anti-mouse IgG (Invitrogen, Carlsbad, CA, USA) and Alex Fluor 594-conjugated anti-rabbit IgG (Invitrogen) were used as secondary antibodies. After being rinsed, the cells were mounted in media containing DAPI. The images acquired via confocal microscopy (Nikon C1) were analysed with ImageJ. Representative high-power fields that showed syntaxin4 or CRACM3 immunofluorescence were segmented from background objects and scored. The fluorescence intensity of a high-power field was divided by the number of cells to determine the fluorescence intensity per cell.

### Detection of Ca^2+^ influx and electrophysiology

Cameleon (pcDNA3-YC4.60-CAAX)-transfected RBL-2H3 cells were directly plated on the plates 24 h before the assay. A series of doses of extracellular Ca^2+^ were applied to the wells. The assay was performed using a fluorometric imaging plate reader (FLIPR) (FlexStation II, Molecular Devices Japan, Tokyo, Japan). The plates were equilibrated to 37 °C for 15 min before the start of the assay and read for a total of 700 s, including an initial 100-s reading window to measure the baseline fluorescence levels before the application of any compound. The plates were read for an additional 600 s after 0.5 μM TG. Calcium signals were read using 440 nm excitation and 530/480 nm emission. The results are presented as the ratio of relative fluorescence units (R.F.U.) (530 nm/480 nm). Because the final concentration of dimethyl sulphoxide in the assay was 1%, vehicle controls of 1% dimethyl sulphoxide were included in each assay plate.

The whole-cell configuration is used to measure the membrane capacitance as a function of time. To determine changes in patch capacitance in the whole-cell configuration, adherent RBL-2H3 cells were plated on Petri dishes 2 d before experiments. A connection between the patch-pipette lumen and the cytosol is made by applying a negative pressure of sufficient magnitude. A thin-wall, Sylgard-coated, relatively large diameter 4- to 6-MΩ patch pipette was filled with an intracellular solution of 150 mM monopotassium glutamate, 6 mM MgCl_2_, 1 mM EGTA, 10 mM Hepes, 0.5 mM inosine triphosphate, and 5 μM guanosine 5′-[3-thio]-triphosphate, pH 7.20. We triggered exocytosis with different [Ca^2+^]_free_: a solution with the indicated free Ca^2+^ concentration was infused into the cell to induce membrane fusion. All free Ca^2+^ values given in this article were calculated with Ca-EGTA Calculator v1.3 using constants from Theo Schoenmakers’ Chelator. Liquid junction potentials were corrected before the configuration was made, and pipette capacitance was electronically maintained using an amplifier (Axopatch 200B Axon Instruments, Foster City, CA, USA). The currents were digitized at 4 kHz with an analogue-digital converter (Digidata 1440A, Axon Instruments, Sunnyvale, CA, USA). Capacitance was recorded simultaneously, and passive cell parameters in the whole-cell configuration were calculated according to previous protocols^25^,^26^.

### Real-time imaging of single nanoparticles in cultured RBL-2H3 cells

A custom-designed upright multi-photon excitation microscopy system (A1RMP, Nikon Corporation, Japan), equipped with a water-immersion high numerical-aperture (NA) objective lens (CFI75 Apo 25xW MP, N.A. = 1.10, Nikon) and InSight DeepSee ultrafast laser source (680–1300 nm, Spectra-Physics K.K., Tokyo, Japan) was set up as described previously^27^. A shortpass filter at 770 nm (FF01-770/SP-25, Semrock, Inc. Rochester, New York, USA), a dichroic mirror at 560 nm (FF560-Di01-25 × 36, Semrock), and a bandpass filter at 617 nm (FF02-617/73, Semrock) were used to capture Qdot fluorescence. The laser power was 5 to 10 mW, and the excitation wavelength was 1200 nm. Images were acquired every 0.25 to 1 s. All imaging experiments were performed at 37 °C.

Qdots were loaded as described previously with minor modifications^11^. Streptavidin-coated Qdots^®^625 (Life Technologies, Eugene, OR, USA) with a diameter of ~18 nm were preincubated at room temperature for 1 h with biotinylated synaptotagmin1 antibodies (Synaptic Systems, Gottingen, Germany). The final concentration of antibody-conjugated Qdots was limited to 0.1 nM for sparse labelling. Next, 2.5 mM DDT and 0.04% casein were added to the PIPES loading buffer to increase the photostability of the Qdots.

After 100 s of additional preincubation at 37 °C, Qdot-loaded RBL-2H3 cells were imaged continuously. Cells were perfused with extracellular solution that contained TG at a final concentration of 1 μM for the stimulation. The perfusion time was ~10 s. The imaging period consisted of 10 s without stimulation followed by 580 s after TG-stimulation.

All acquired images were saved as AVI files and analysed off-line with Particle Track and Analysis plugins (The Institute of Scientific and Industrial Research, Osaka University, Japan) for ImageJ according to an established protocol. The regions of interest (ROIs) were defined to have a diameter of 9 pixels (~1.3 μm). The average intensities of Qdot photoluminescence in the ROIs were analysed in a timely manner and exported as text files. All intensity values are shown after background subtraction. Traces whose amplitude fell within the range of 200–1300 a.u. were analysed. Trace smoothing was performed by binomial smoothing methods using GRAMS/AI version 9 software (Thermo Scientific, Waltham, MA, USA). The upticks and downsteps were searched independently for individual fusion events. Positive deflections were defined as events whose highpass-filtered trace crossed a threshold ~2.5 times the standard deviation of the pre-stimulus intensity (2-s pre-stimulation baseline). The onset of an uptick or a downstep was determined as the time point when the intensity reached 25% of its peak or final level, respectively. Negative deflections were analysed only if their amplitude fell within the range of 20–100 a.u.

### Statistical analysis

All experiments were designed in a completely randomized multifactorial format. The results are expressed as the mean ± s.e.m. Pair-wise comparisons were performed using a two-tailed Student’s *t*-test. Chi-squared tests were used to evaluate the statistical significance of distributions of fusion events. Two-factor factorial ANOVA followed by the Scheffé F-test was used to compare the dose-response curves of increased *Cm* to [Ca^2+^]_free_ to account for the unequal variance. *P* values < 0.05 were considered significant.

## Additional Information

**How to cite this article**: Liu, S. *et al*. CRACM3 regulates the stability of non-excitable exocytotic vesicle fusion pores in a Ca^2+^-independent manner via molecular interaction with syntaxin4. *Sci. Rep.* **6**, 28133; doi: 10.1038/srep28133 (2016).

## Supplementary Material

Supplementary Information

## Figures and Tables

**Table 1:** The sequences of siRNA.

Target	Sequences (5′-3′)	Sequences (5′-3′)
Syntaxin4 (NM031125) cocktail	GGAAGAAGCUGAUGAGAAUTT	AUUCUCAUCAGCUUCUUCCTT
	CAAGAUAGCGCUAGAGAAUTT	AUUCUCUAGCGCUAUCUUGTT
	CACCAUAACCGUUGGAUAATT	UUAUCCAACGGUUAUGGUGTT
CRACM3 (NM001014024) cocktail	GUUUAUGGCCUUUGCCCUA	
	GCGGCUACCUCGACCUUAU	
	UGGAGAGCGAUCAUGAAUA	
	ACCAACGACUCCACCGAUA	
Negative control duplex	ATCCGCGCGATAGTACGTA	
	TTACGCGTAGCGTAATAGG	
	TATTCGCGCGTATAGCGGT	
