# Supplementary material for: CRACM3 regulates the stability of non-excitable exocytotic vesicle fusion pores in a Ca2+-independent manner via molecular interaction with syntaxin4
Source: Sci Rep. 2016 Jun 15;6:28133. doi: 10.1038/srep28133 (PMC4908399; doi:10.1038/srep28133)
Supplement: Supplementary Information [file srep28133-s1.pdf]

# Supplementary information

## CRACM3 regulates the stability of non-excitable exocytotic vesicle fusion pores in a $Ca^{2+}$-independent manner via molecular interaction with syntaxin4

Shuang Liu, Muhammad Novrizal Abdi Sahid, Takemasa Erika, Takeshi Kiyoi, Miyuki Kuno, Yusuke Oshima , Kazutaka Maeyama

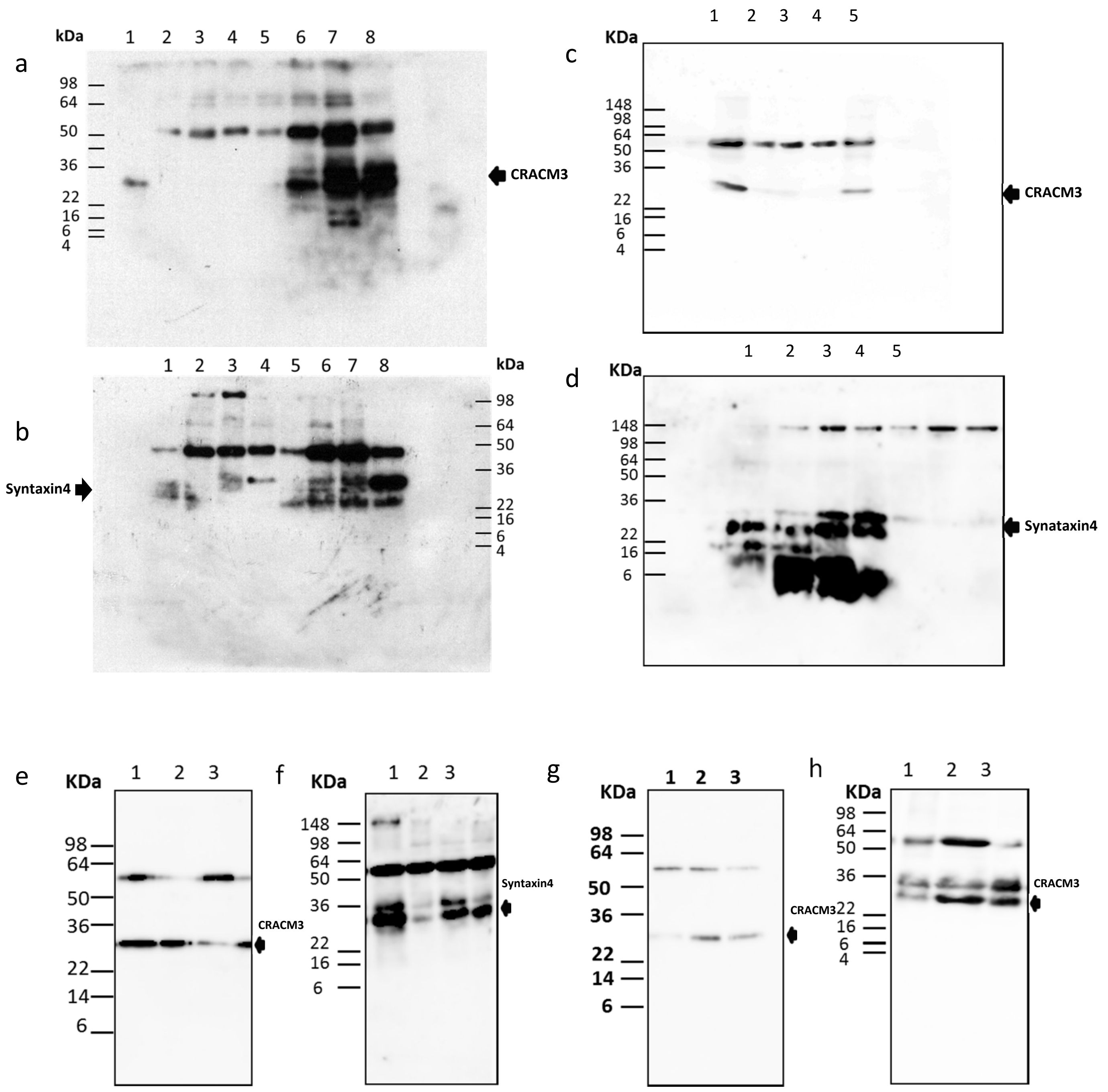


**Supplementary fig. 1 (a)** Full-length blots of CRACM3 protein levels were measured by western blotting after immunoprecipitation using specific antibodies for CRACM1, CRACM2, CRACM3, and syntaxin4. The expression of CRACM3 in non-stimulated control cells (lane 1: CRACM1-immunoprecipitation; lane 2: CRACM2-immunoprecipitation; lane 3: CRACM3-immunoprecipitation; lane 4: synatxin4-immunoprecipitation) and TG-stimulated cells (lane 5: CRACM1-immunoprecipitation; lane 6: CRACM2-immunoprecipitation; lane 7: CRACM3-immunoprecipitation; lane 8: synatxin4-immunoprecipitation). **(b)** Full-length blots of syntaxin4 protein levels were measured by western blotting after immunoprecipitation using specific antibodies for CRACM1, CRACM2, CRACM3, and syntaxin4. The expression of syntaxin4 in non-stimulated control cells (lane 1: CRACM1-immunoprecipitation; lane 2: CRACM2-immunoprecipitation; lane 3: CRACM3-immunoprecipitation; lane 4: synatxin4-immunoprecipitation) and TG-stimulated cells (lane 5: CRACM1-immunoprecipitation; lane 6: CRACM2-immunoprecipitation; lane 7: CRACM3-immunoprecipitation; lane 8: synatxin4-immunoprecipitation). **(c)** Full-length blots of CRACM3 protein, which was co-immunoprecipitated using the His-tag in rSx4-His-wild- (lane 1), rSx4-His-mTM- (lane 2), rSx4-His-mH3- (lane 3), rSx4-His-mIIS- (lane 4), and rSx4-His-mNp-transfected cells (lane 5), was detected using western blotting. **(d)** Full-length blots of syntaxin4 protein, which was co-immunoprecipitated with His-tag in rM3-His wild- (lane 1), rM3-His-mNTD- (lane 2), rM3-His-mL1- (lane 3), rM-His-mL2- (lane 4), rM3-His-mL3- (lane 5), and rM3-His-mCTD-transfected cells (lane 6), was detected by western blotting. **(e)** Full-length blots of the protein levels of CRACM3 3 d post transfection of siRNA were detected by western blotting following siRNA-mediated protein suppression. CRACM3 expression in NCsiRNA- (lane 1), Syn4siRNA- (lane 2), and M3siRNA-transfected cells (lane 3). **(f)** Full-length blots of the protein levels of syntaxin4 3 d post transfection of siRNA were detected by western blotting following siRNA-mediated protein suppression. Syntaxin4 expression in NCsiRNA- (lane 1), Syn4siRNA- (lane 2), and M3siRNA-transfected cells (lane 3). **(g)** Full-length blots of CRACM3 expression in total cells lysates of control cells (lane 1), rM3-His-wild-transfected cells (lane 2), and rM3-wild-transfected cells (lane 3). **(h)** Full-length blots of CRACM3 expression detected by western blotting followed by syntaxin4 immunoprecipitation in total cells lysates of control cells (lane 1), rM3-His-wild-transfected cells (lane 2), and rM3-wild-transfected cells (lane 3).

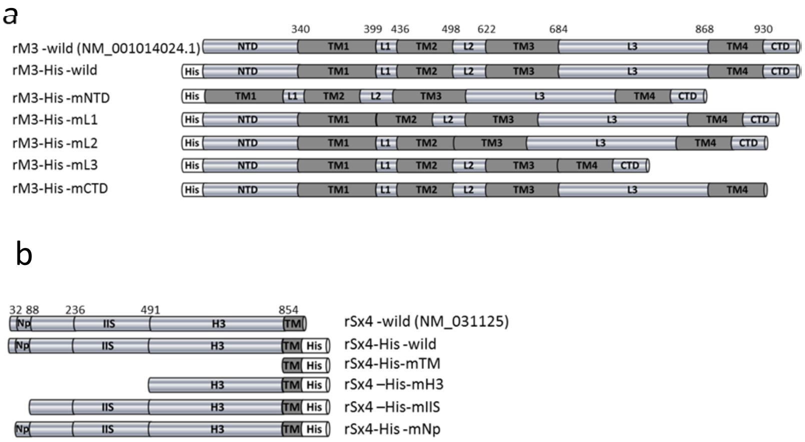


**Supplementary fig. 2 (a)** Schematic showing different forms of His-tagged CRACM3 truncations. **(b)** Schematic showing different forms of His-tagged syntaxin4 mutants.

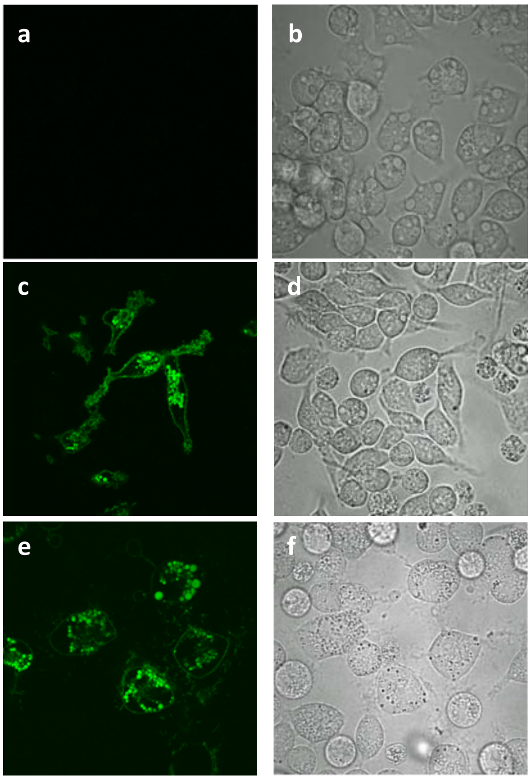


**Supplementary fig. 3** The syntaxin4 mutants' expression in transfected RBL-2H3 cells. We prepared GFP-tagged syntaxin4 truncations and transfected RBL-2H3 cells to confirm the location of the syntaxin4 mutants' expression. The syntaxin4 truncations without N-peptide that were used in this experiment are the H3-including truncation (rSx4-GFP-mH3) or an IIS-including truncation (rSx4-GFP-mIIS). Live cell images are shown for the non-transfected cells (a, b), the cells transfected with rSx4-GFP-mH3 (c, d), and rSx4-GFP-mIIS (e, f).

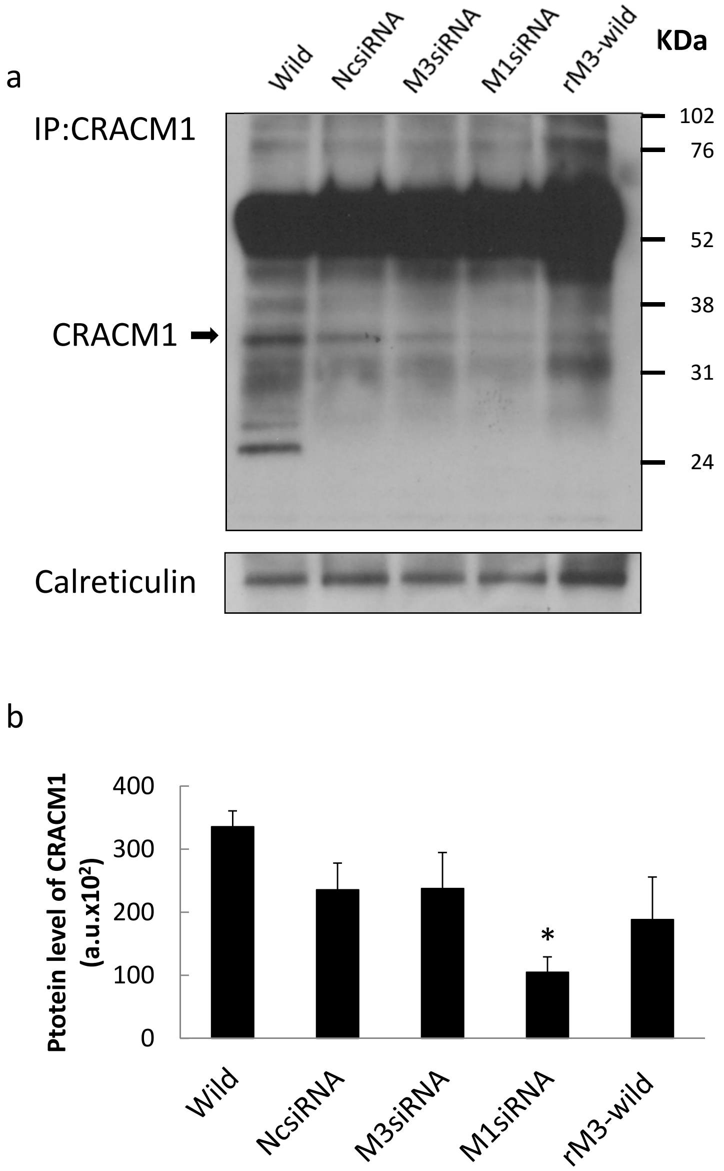


**Supplementary fig. 4** CRACM1 protein level that was detected by western blotting after CRACM1 was precipitated from cell lysates using a CRACM1-specific antibody. Upper: CRACM1 expression from wild type cells, M1siRNA-, M3siRNA-, NcsiRNA- , and rM3-wild-transfected cells. Lower: CRACM1 expression was normalized to calreticulin levels in total cell extracts. (b) CRACM1 protein expressions in RBL-2H3 cells (a.u., arbitrary units, * *P*<0.05, n=3 ).

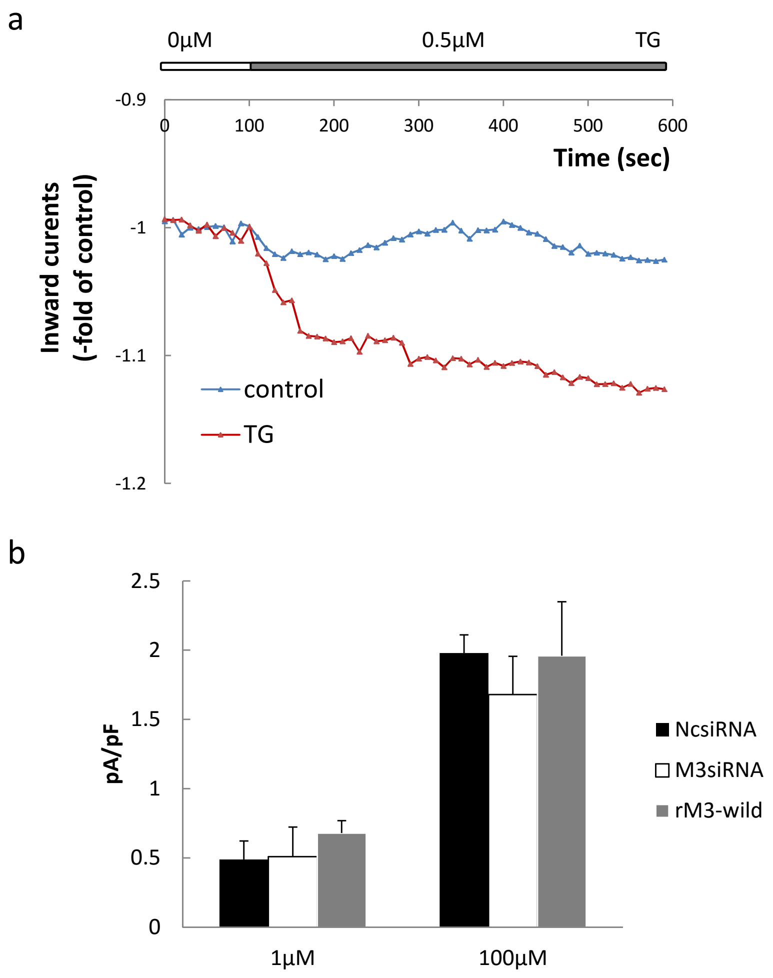


**Supplementary fig. 5** Whole-cell patch-clamp recordings to measure CRAC-like currents in M3siRNA-, NCsiRNA-, and rM3-wild-transfected cells treated with 1 μM and 100 μM extracellular $Ca^{2+}$.Under whole-cell conditions, whole-cell currents were elicited by 1-s-long voltage ramps from -100 to +10mV every 10s. Prior to the stimulation, 10-20 voltage ramps were applied to obtain base line current values as controls. All current values are shown after leak subtraction. All experiments were conducted at 25°C. (a) Typical time course of CRAC-like currents activation. Amplitude of inward currents recorded at -80mV are expressed as the fold increase over n pre-stimulation control values in the cells for each group. The CRAC – like currents were defined as the difference between the control and post-stimulation currents. (b) The maximal response induced by TG in M3siRNA-, NCsiRNA-, and rM3-wild-transfected cells treated . In each cell, the average of 10 traces in the control was subtracted from the mean of 10 traces around the maximal response induced by TG with the applications of 1μM or 100μM extracellular $Ca^{2+}$. The average of 10-12 cells was obtained for each group. The data are present as mean±s.e.m..

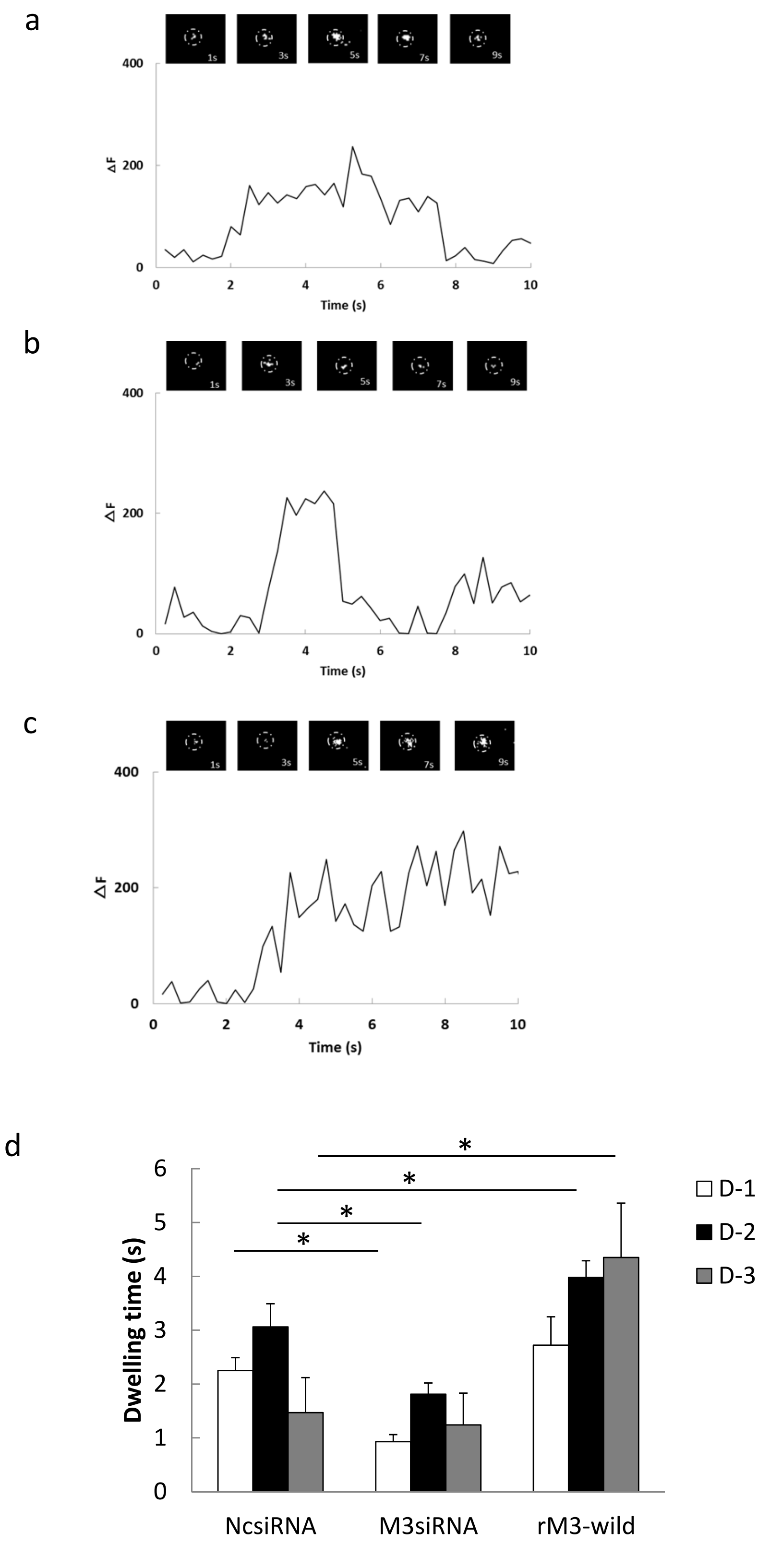


**Supplementary fig. 6** The typical intensity trace and parallel images of Qdot photoluminescence in ncsiRNA-, M3siRNA-, and rM3-wild-transfected cells. The Qdot-loaded plasma membrane exhibited different patterns of photoluminescence upon TG-stimulation and images of a Qdot cluster at multiple time points in ncsiRNA- **(a)**, M3siRNA- **(b**), and rM2-wild-transfected **(c)** RBL-2H3 cells. **(d)** The dwell times of the initial state (D-1), the full-open state (D-2), and the closed state (D-3) in ncsiRNA-, M3siRNA -, and rM2-wild-transfected RBL-2H3 cells. The average of photoluminescence for 250 single stimulus presentations in of each group. *$P$<0.05, The data are present as mean±s.e.m..

```
               10        20           30             40
STX3   MKDRLEQLKAKQLTQDDDTDEVEIAI---DNTAFM----DEFFSEIEETR
       :.:: ..:.  .  .::. :::..:.   ...: .    ::::....  :
STX4   MRDRTHELRQGDNISDDE-DEVRVALVVHSGAARLSSPDDEFFQKVQTIR
               10         20         30        40
```

**Supplementary fig.7** We compared the sequences of 40-amino-acid-long segments within the cytoplasmic C-termini of syntaxin3 and syntaxin4, which should contain the predicted N-peptide region. Synatxin3 shares only 32.0% amino acid sequence identity with syntaxin4.
